# Canine lymphoma in Croatia: a fourteen-year retrospective study

**DOI:** 10.1186/s12917-025-04634-x

**Published:** 2025-03-17

**Authors:** Vida Eraghi, Lidija Medven Zagradišnik, Mavro Matasović, Dunja Vlahović, Doroteja Huber, Andrea Gudan Kurilj, Ivan-Conrado Šoštarić-Zuckermann, Branka Artuković, Ivana Mihoković Buhin, Iva Ciprić, Marko Hohšteter

**Affiliations:** 1 Department of Veterinary Pathology, Faculty of Veterinary Medicine, University of Zagreb, Zagreb, Croatia; 2 Vet Point, Veterinary Practice, Zagreb, Croatia; 3 Bioinstitut d.o.o, Čakovec, Croatia

**Keywords:** Canine lymphoma, Croatia, Retrospective study, Cytology, Immunohistochemistry, Immunocytochemistry

## Abstract

**Background:**

Lymphoma is the most prevalent hematopoietic system tumor in dogs and holds significant clinical importance in veterinary medicine. However, the epidemiology of canine lymphoma in Croatia remains understudied. This retrospective study aims to describe the predominant lymphoma types in this population over 14 years and evaluate associations with sex, breed, and age.

**Results:**

Among 28,681 canine cases referred to the Department of Veterinary Pathology, University of Zagreb, from 2009 to 2023, a total of 374 cases (1.30%) were diagnosed with lymphoma. Of these, 282 cases (75.40%) were purebred dogs, with the most affected breeds being Golden Retrievers (23, 6.15%), Labrador Retrievers (23, 6.15%), German Shepherds (14, 3.74%), and Boxers (14, 3.74%). Bullmastiffs (8.14%) had the highest number of lymphoma diagnoses among the referred breeds, followed by Airedale Terriers (6.67%) and German Shepherds (5.09%). The Maltese, though a popular breed, showed a low lymphoma rate of 0.40%, indicating no notable predisposition. Multicentric lymphoma (59, 53.64%) was the most common anatomical classification, followed by cutaneous (33, 30.00%) and alimentary lymphoma (13, 11.82%). The mean age at diagnosis was 8.27 ± 3.07 years, with most cases occurring between 5 and 10 years (207, 55.35%). Males (216, 57.75%) were more frequently affected than females (158, 42.24%), except among Golden Retrievers, where 69.56% of cases were female.

**Conclusion:**

Our study reveals that the distribution of dogs with lymphomas, including type and anatomical classification, in terms of breed, sex and age, is consistent with existing literature, except in the cases of Golden Retrievers, Basset Hounds, and Scottish Terriers. Further molecular and environmental studies are recommended.

## Background

One of the most common malignancies in dogs is lymphoma [1, 2]. The dogs with single, regional or systemic lymphadenomegaly who are referred to the clinician, will be suspected to lymphoma [3, 4]. In this malignancy the lymphatic system cells originate from clonal expansion of neoplastic cells of the lymphoid lineage in primary or peripheral lymphoid organs, and can subsequently spread to other lymphocyte-accessible tissues [4]. Lymphomas can be classified into different types and subtypes based on their anatomical location, type of neoplastic cells, their immunophenotype, and other cytological and histological characteristics [4]. According to Vail and Young [5], the anatomical classification includes multicentric lymphoma, mediastinal (or thymic) lymphoma, alimentary lymphoma, extranodal lymphoma (involving primarily affected tissues outside the lymphatic system), and cutaneous lymphoma. To achieve a more precise classification of lymphomas and to identify whether they fall into the categories of T-cell, B-cell, or other lymphoma types, it is necessary to employ immunophenotyping techniques, such as immunocytochemical (ICC) or immunohistochemical (IHC) staining [6].

Lymphomas in dogs are generally fatal, although they can be kept under control for some time and dogs can be maintained alive with various chemotherapy protocols [3, 7, 8]. Timely diagnosis and typing of lymphoma in dogs allow for determining prognosis and selecting the appropriate therapy, greatly extending the dog’s survival time [7, 8].

Cytological, histological, and immunochemical diagnosis of lymphoma conducted on referred samples of cytological aspirates from enlarged lymph nodes or suspicious altered tissues remains the gold standard for lymphoma diagnosis, despite the increasing use of molecular diagnostic methods [9–13]. Using cytology is a reliable method for the diagnosis of canine lymphoma. This method could be sufficient in the diagnosis process, and it is also a safe and cost-effective method [3, 9–14]. Fine-needle aspiration (FNA) cytology is a practical and fairly sensitive method for diagnosing high-grade lymphomas and is commonly used [1, 15]. However, FNA cytology is not satisfactory for reliable diagnosis of low-grade lymphomas or atypical forms of lymphoid neoplasms. In such cases, histopathological examination of biopsies is necessary [6].

In this study, we compile data pertaining to canine lymphoma cases diagnosed at the Department of Veterinary Pathology, University of Zagreb, over the past 14 years. The objective of this retrospective study is to enhance our understanding of the predominant lymphoma types in this population and assess the associations with breed, sex and age. These insights are valuable for refining the diagnostic process and treatment strategies.

## Results

**Fig. 1:**
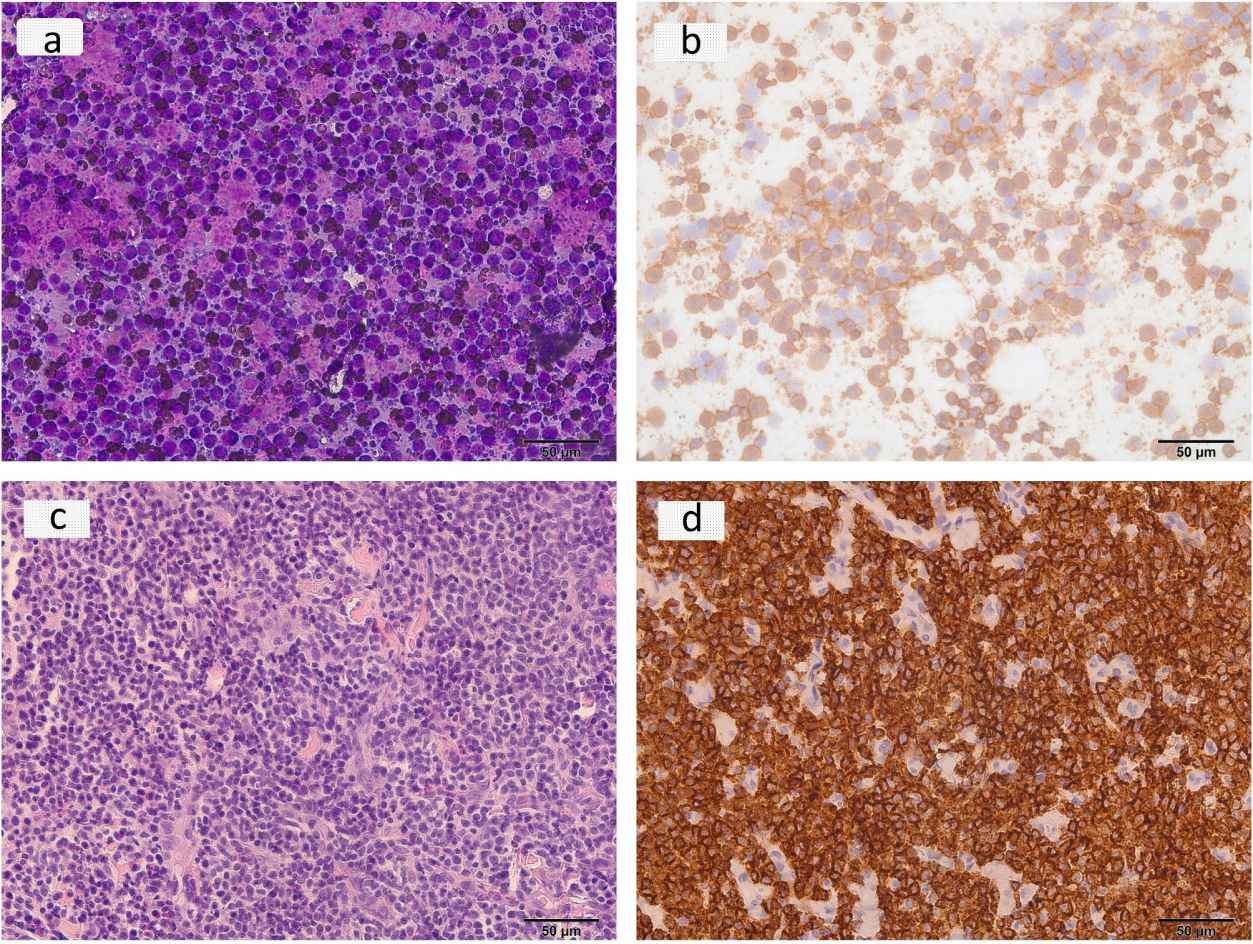
Microscopic images showing canine lymphoma: **a** Fine-needle aspiration biopsy (FNA) from a lymph node reveals intricate details of B-cell lymphoma using Modified Giemsa Stain (40x). **b** Immunocytochemistry of the FNA biopsy from the lymph node demonstrates CD20 positivity in B-cell lymphoma (40x). **c** Histopathological examination of the lymph node sample unveils distinctive features indicative of T-cell lymphoma, stained with H&E (40x). **d** Immunohistochemistry image showcases CD3 expression in T-cell lymphoma (40x)

From 2009 to August 2023, the Department of Veterinary Pathology received a total of 28,681 referrals for canine cases. Among these cases, 20,428 (71.22%) were of purebred lineage, with the three most prevalent purebred breeds being Labrador Retriever (1577, 7.72%), Maltese (1235, 6.04%), and Golden Retriever (1095, 5.36%). Out of the entire population, 374 dogs (1.30%) received a diagnosis of canine lymphoma. Lymphoma was diagnosed through necropsy, histology, cytology, and/or immunochemistry. Figure 1 includes representative images of positive cases, illustrating diagnostic slides from these methods (Fig. 1).

### Sampled tissues

In the diagnostic process for lymphoma, a variety of tissue samples were collected, with lymph node aspiration being the most frequently submitted sample for examination. After lymph node aspirations in terms of frequency were lymph node and skin biopsies, followed by biopsies of the gastrointestinal tract, spleen, and liver.

### Anatomical classification of lymphoma

The anatomical classification, based on Vail and Young [5], categorized lymphoma cases into five groups: multicentric lymphoma, cutaneous lymphoma, alimentary lymphoma, mediastinal lymphoma, and extranodal lymphoma. Anatomical classification data were available for 110 dogs (29.41%) of the confirmed lymphoma cases. Among these cases, the most prevalent anatomical subtype of lymphoma was multicentric lymphoma (59, 53.64%), followed by cutaneous lymphoma (33, 30.00%), and alimentary lymphoma (13, 11.82%). The least prevalent subtype was extranodal lymphoma. Statistical analysis using the Chi-square test for independence revealed significant differences between multicentric and cutaneous lymphoma (p value = 0.02) as well as between multicentric and alimentary lymphoma (p value = 0.004). However, the difference between cutaneous and alimentary lymphoma was not statistically significant (p value > 0.05). The mean ages for each lymphoma subtype ranged from 8.31 ± 3.00 years (cutaneous lymphoma) to 9.46 ± 2.82 years (alimentary lymphoma), except for mediastinal lymphoma and extranodal lymphoma, which had lower mean ages of 5.33 ± 3.09 years and 3.50 ± 1.50 years, respectively. Notably, cutaneous lymphoma was the predominant anatomical classification for Golden Retrievers, accounting for 62.5% of cases. In contrast, Labrador Retrievers demonstrated an equal frequency of 50% for both multicentric and alimentary lymphoma. Among non-purebred dogs, multicentric lymphoma was the most common subtype, representing 63.6% of cases (Table 1).

**Table 1:** Canine lymphoma descriptive analysis by anatomical form - frequency, percentage, mean and median age, minimum-maximum values

	*N*	%	Mean age (SD)	Median age (min-max)
Multicentric	59	53.64%	8.37 (3.21)	8 (1–15)
Cutaneous	33	30.00%	8.31 (3.00)	8 (1–14)
Alimentary	13	11.82%	9.46 (2.82)	10 (2–13)
Mediastinal	3	2.73%	5.33 (3.09)	-
Extranodal	2	0.91%	3.5 (1.5)	-

For entities with less/equal three cases, the median age was not calculated

### Age

The age range of dogs referred to the department and diagnosed with lymphoma spanned from less than one year to seventeen years, with a mean age of 8.27 ± 3.07. The number of dogs diagnosed with lymphoma showed an increasing trend from birth to eight years of age, followed by a decline up to seventeen years of age. Notably, the most common age group among dogs diagnosed with lymphoma falls within the 5–10 years old range, accounting for 55.8% of cases, followed by more than 10 years old (33%), and less than 5 years old (11.2%). Based on Chi-square test for independence, a significant difference in the distribution of lymphoma cases among the age groups was observed (p value < 0.05).

In all dog breeds, most of lymphoma cases occurred within the age range of 5 to 10 years. Nevertheless, an intriguing pattern unfolds when examining Golden Retrievers, with a striking 73.9% of lymphoma cases in this breed occurring in dogs over 10 years of age. The highest mean age was observed among Border Collies (11.33 ± 0.47), followed by Maltese (10.00 ± 3.16) and non-pure breeds (9.09 ± 2.91), while the lowest mean age was found in Dobermans (5.80 ± 2.29). Based on Spearman’s rank correlation test, a strong correlation between age and specific breeds was not observed. For Golden Retrievers, a relatively high correlation coefficient (ρ = 0.794) was found; however, the p-value (0.059) exceeds the significance threshold, indicating that this correlation is not statistically significant.

### Sex

Among dogs diagnosed with lymphoma, 216 (57.75%) were male, while 158 (42.24%) were female. The average age for male dogs with lymphoma was 8.06 ± 3.13 years, while for female dogs, it was 8.60 ± 2.95 years. Notably, in contrast to other breeds, a significant majority (69.56%) of Golden Retrievers diagnosed with lymphoma were female. Between the ages of 1–5 years and 5–10 years, male dogs outnumber females (p value = 0.06). In the third age group, the number of males and females was equal.

### Breed

Among the purebred dogs referred to our department, 272 cases (1.33%) were diagnosed with lymphoma, compared to 92 cases (1.11%) of non-purebred dogs. Of the total lymphoma cases, 74.73% were found in purebred dogs, while the remaining 25.27% occurred in non-purebred dogs. Among purebred dogs, the most prevalent breeds with lymphoma were Golden Retrievers (23, 6.15%), Labrador Retrievers (23, 6.15%), German Shepherd (14, 3.74%) and Boxers (14, 3.74%). Additionally, we calculated the proportion of canine lymphoma for specific breeds who referred to our department during this period: Bullmastif (8.14%), Airedale Terriers (6.67%), German Shepherd (5.09%), Shar pei (4.65%), Doberman (2.47%), Rottweilers (2.24%) and Golden Retrievers (2.10%) (Table 2).

When focusing on the three most common purebred breeds referred to our department, it’s noteworthy that Maltese, despite being a fairly popular breed, did not exhibit a particularly high predisposition to lymphoma compared to other breeds, with a rate of 0.40%. In contrast, the rates were 2.10% for Golden Retrievers and 1.46% for Labrador Retrievers.

**Table 2:** Distribution of lymphoma cases by breed referrals to the department

	Number of Total Cases	Number of Lymphoma Cases	%
Labrador Retriever	1577	23	1.46%
Maltse	1235	5	0.40%
Golden Retriever	1095	23	2.10%
Bulldog	763	9	1.18%
Poodle	693	2	0.29%
Boxer	690	14	2.03%
Shih-Tzu	570	7	1.23%
Rottweiler	357	8	2.24%
Scottish Terrier	341	1	0.29%
Beagle	333	7	2.10%
German Shepherd	275	14	5.09%
Doberman	242	6	2.47%
Shar pei	86	4	4.65%
Bullmastif	86	7	8.14%
Basset Hound	56	0	0
Airedale Terriers	45	3	6.67%
Non-Purebred	8253	92	1.11%

### Immunotyping of lymphoma

Among all lymphoma cases, 113 cases (30.21%) underwent immunophenotyping using immunocytochemistry (ICC) and/or immunohistochemistry (IHC). Within this subset, 65 cases (57.52%) were classified as B-cell lymphoma, and 47 cases (41.82%) as T-cell lymphoma. Notably, among the cases of B-cell lymphoma, 7 (10.77%) were identified as T-cell-rich B-cell lymphoma. A majority of cases in both B-cell and T-cell lymphoma groups belonged to purebred dogs.

The mean age for dogs with B-cell lymphoma was 8.23 ± 2.75 years, while those with T-cell lymphoma had a slightly higher average age of 8.58 ± 2.78 years. Consistent with the findings among all dogs diagnosed with lymphomas in this study, males were more prevalent in both the T-cell and B-cell lymphoma groups.

## Discussion

In our retrospective observational study, we sought to unveil the predominant lymphoma types within this population and assess the potential influence of several associations, including breed, sex and age. Our study presents a significant advantage in the comprehensive and robust nature of the data collected. The compilation of canine lymphoma cases spanning a 14-year period not only offers a substantial sample size but also provides a unique long-term perspective on the disease. By analyzing a large set of cases, our research contributes to a deeper understanding of the disease’s dynamics, offering valuable insights that can guide clinical practice and research strategies.

Lymph node aspiration, lymph node biopsies, and skin biopsies were the most frequently submitted samples for the diagnosis of lymphoma during these years, consistent with findings from previous research [16]. The predominance of multicentric lymphomas is in line with existing literature, which has previously identified them as the most prevalent type in dogs [6, 17, 18]. The lowest number of diagnoses was associated with extranodal lymphoma, aligning with the findings in the research conducted by Pinelo et al. [18].

As previously reported by Teske [19] and Edwards et al. [20], the occurrence of lymphoma in dogs exhibited a relatively consistent increase until the eighth year, followed by a decline. The mean age in our study, which was 8.27 ± 3.07, closely resembled the findings in other research [17, 21–25].

Consistent with findings in other studies, a higher proportion of lymphoma cases were male [17, 23, 26, 27]. Across all categories in our study, the proportion of males exceeded that of females, except in the case of Golden Retrievers, where the majority were female. This differs from another study on Golden Retrievers, where males were more frequently diagnosed with cancer. This article suggests that neutering Golden Retrievers may increase the likelihood of cancer, including lymphoma, and it’s possible that a significant portion of our cases were neutered [28, 29].

Distinguishing the immunophenotypes of B or T lymphomas has evolved into a crucial method for classification and prognosis, as noted by Ponce et al. [17, 30] and Valli et al. [31]. In this study, B-cell neoplasia constituted 65 (57.52%) of all diagnosed cases for which the immunophenotype was determined. This higher representation of B-cell lymphomas compared to T-cell lymphomas aligns with established knowledge in the field [18, 32, 33].

Regarding breeds, the majority of dogs diagnosed with lymphoma belonged to purebred dogs, accounting for 74.73% of the cases. The increased predisposition of purebred dogs to develop lymphoma has been previously documented in the literature and is often attributed to a more limited genetic pool and an elevated prevalence of genetic abnormalities [6]. This was followed by non-purebred dogs, which represented 25.27% of the cases, consistent with findings in other studies [18, 25].

Among purebred dogs, the most frequently encountered breeds were Golden Retriever, Labrador Retriever, German Shepherd, and Boxer, consistent with previous studies that examined breed predisposition to cancer in dogs [4, 34]. These articles primarily focused on examining the most prevalent lymphoma cases. In contrast, our study examined the most frequently observed cases and evaluated the occurrence of lymphoma within each specific breed among all cases referred to our department. Certainly, this method cannot substitute for the precise calculation of lymphoma incidence rates in specific dog breeds over time. Nonetheless, it can function as a preliminary, rough estimate and provide valuable guidance for future comprehensive studies in the field of veterinary epidemiology that may be undertaken in Croatia. Among dogs referred to our department during this period, the three breeds with the highest occurrence of lymphoma are Bullmastiff, Airedale Terriers, and German Shepherds. Conversely, the lowest occurrence is observed in breeds such as Basset Hound, Poodle, Scottish Terrier and Maltese. It’s worth noting that our findings align with previous studies, except for Basset Hound and Scottish Terrier, as reported by Bennett et al. [35]. Interestingly, our data deviates from other articles that suggest an increased risk of lymphoma in Basset Hound and Scottish Terrier breeds. It’s crucial to acknowledge that variations in breed predisposition observed between studies may be attributed to discrepancies in breed popularity and population sizes across different countries. This issue can only be definitively resolved with the implementation of a comprehensive animal census.

## Conclusions

In conclusion, our study has shed light on the occurrence of lymphoma among dogs referred to our department. These findings not only contribute to our understanding of lymphoma in canine populations but also highlight the importance of breed considerations in disease epidemiology. As we continue to expand our knowledge in this field, these insights can guide future research and inform targeted interventions for the well-being of our canine companions. Environmental factors, such as exposure to pollutants, pesticides, and urbanization, may play a role in the development of canine lymphoma and should be further explored to understand their impact. Additionally, we advocate for molecular investigations to delve deeper into breed-specific genetic factors, enabling the development of precise prevention and intervention strategies tailored to the unique needs of individual breeds.

## Methods

### Data collection

This study is a hospital-based retrospective analysis focused on diagnosed cases of canine lymphoma, along with associated data on breed, sex, and age. The cases were collected from the Department of Veterinary Pathology, University of Zagreb, covering the period from 2009 to August 2023.

Additionally, we compiled data on the population numbers and breeds of all dogs referred to our department throughout this timeframe due to the limited availability of comprehensive population data for the studied animal species within our specific geographic region.

### Diagnosis of lymphoma

The diagnosis of lymphoma was conducted using cytology, histopathology, immunocytochemistry (ICC), and/or immunohistochemistry (IHC). Immunophenotyping of canine lymphomas was carried out using specific antibodies to identify cell surface markers and classify lymphoma subtypes. Monoclonal antibodies were used for CD3 (Dako) as a marker for T-cell lymphoma, while CD20 (Invitrogen) and served as markers for B-cell lymphoma. For ICC/IHC procedures, antigen retrieval was performed using heat-induced epitope retrieval with Dako Target Retrieval Solution (pH 9 for CD3, pH 6 for CD20). Staining was conducted on a DAKO Autostainer Plus using CD3 (1:50) and CD20 (1:300) with EnVision HRP detection and DAB chromogen.

The classification of lymphomas was performed as B- or T-cell types when at least 70% of neoplastic cells were labeled with their respective immunomarkers, or as null (O) in cases where no immunolabeling was observed [17, 18].

### Statistical analysis

Descriptive statistics were used to summarize data. Chi-square test for independence were conducted to assess associations between categorical variables. Spearman’s rank correlation test were done for checking the correlation between age and breeds. Statistical significance was considered for a p-value less than 0.05. The analyses were performed using IBM SPSS Statistics software (Version 22).

## Data Availability

All data supporting the conclusions of this article are included within the article.
